# Ethical standards for medical research in the Israeli military - review of the changes in the last decade

**DOI:** 10.1186/s13584-016-0113-4

**Published:** 2016-12-01

**Authors:** Ayal Hassidim, Raeed Kayouf, Nirit Yavnai, Naomi Panush, David Dagan, Tarif Bader, Michael Hartal

**Affiliations:** 1 Israel Defense Forces Medical Corps, Ramat Gan, Israel; 2 Department of Plastic and Reconstructive Surgery, Hadassah-Hebrew University Medical Center, Jerusalem, Israel; 3 Department of Military Medicine, Faculty of Medicine, Hebrew University of Jerusalem, Jerusalem, Israel

**Keywords:** Institutional review board, Ethics committees, Human subject research, Military, IDF

## Abstract

**Background:**

The Israel Defense Forces Medical Corps (IDF MC) institutional review board (IRB) is one of approximately 50 IRBs active in Israel. In addition to routine IRB considerations it must also address in its deliberations specific safeguards in place in the IDF to protect research volunteers in the military environment. In this report, we present the characteristics of the IDF IRB, including the unique circumstances that led to a 2008 change in the pre-IRB advisory and preparatory process (APP). We also present quantitative data on the IRB’s throughput and outcomes, in order to provide a benchmark for other IRBs.

**Methods:**

We reviewed all relevant IDF regulations, both historical and current, pertaining to the structure, activity and oversight of the IRB and of medical research conducted in the IDF. Additionally, we analyzed the ethical review process for all research proposals submitted to the IDF APP between January 1, 2013 and December 31, 2015.

**Results:**

In 2008 the IDF implemented several major changes which have had a substantial impact on the ethical regulation of military medical research. The period following these changes has seen a rise in the number of research proposals submitted to the IDF IRB annually. During the years 2013–2015, 377 research proposals entered the APP, of which 329 were deemed appropriate for IRB deliberation. Eight study protocols were granted waivers, 19 were rejected, and the remaining 302 were authorized. Overall, 345 of the 377 research proposals submitted (92 %) were ultimately cleared for execution; 310 of 329 proposals (94 %) deliberated by the IRB were authorized. The IRB required protocol revisions for 47 % of the research proposals, one-third of which were revisions directly associated with military-specific ethical precautions.

**Conclusions:**

Guided by the principles of protecting personal autonomy in the complex military setting, the IDF has implemented several unique measures aimed at maintaining the highest ethical standards in medical research. By sharing research approval process data similar to those presented here, medical institutions can help build and support a peer-based benchmarking process through which individual IRBs can appraise their own processes and approval rates.

## Background

In August 1947, a US military court reached a decision on the Nuremberg Trials of doctors involved in conducting human experiments in the Nazi concentration camps. In their decision, the justices cited 10 points, later termed the Nuremberg Code, that set the standard for human subject medical research to this day [1]. The Nuremberg Code addresses issues such as informed consent, avoidance of unnecessary pain and suffering, and maintaining the subject’s right to cease participation in the study at any time. This code has since gained substantial standing in the field of medical ethics, yet it has never become codified in law. The Geneva declaration of 1948 [2] further underscored the physician’s responsibilities towards his patients and the guaranteeing of his health and well-being, while only in 1964, during the 18th meeting of the World Medical Association, was a declaration issued specifically addressing the guiding principles of human research [3]. This “Helsinki Declaration” underwent several revisions over time, until the concept of an independent institutional review board (IRB) was introduced in 1975. In 1981, the requirement of an IRB was included in US federal law [4].

**Fig. 1:**
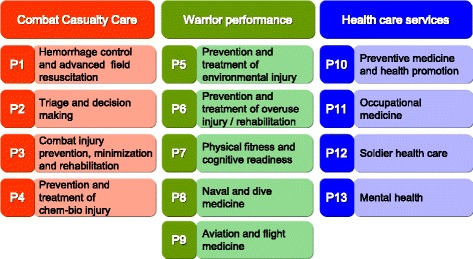
IDF MC 13 permanent research programs

In Israel today, the statutory status of the IRB is rooted in a public health regulations dating from 1980 [5]. Medical institutions in Israel, such as hospitals and the IDF, maintain IRBs. Recent estimates place the number of active IRBs in Israel today at 53 (N.P. - personal communication), including that of the IDF MC. The IDF’s IRB supports an active and robust research effort, which includes 13 permanent research programs and hundreds of individual research projects at any point in time (Fig. 1).

Medical research in the IDF dates back to the 1950s, although many of the results of these early studies were not published in the scientific literature. Throughout the 1980s and early 1990s military medical research experienced a rapid growth in the IDF, and more of the results appeared in scholarly medical journals. Common fields of interest for military medical researchers have routinely included combat casualty care (such as hemorrhage control and triage tools), warrior physiology (such as heat and cold injury, overuse injury and hypobaric and hyperbaric medicine) and soldier health (such as preventive medicine, occupational health and military psychiatry).

Most IDF research subjects, mainly military personnel, are intrinsically and fundamentally different than their civilian counterparts. The majority of IDF soldiers are aged 18–21 years and have only recently reached the age of legal adulthood. Additionally, they serve in a highly hierarchical setting, are acutely susceptible to peer pressure, and are strongly influenced by, and dependent on, their superiors. Due to these constrictions, some soldiers may feel compelled to volunteer for research studies if asked to do so, due to concerns that non-participation might result in negative action by commanders. Furthermore, soldiers are not at liberty to choose their own medical care givers, and military physicians might find themselves in a conflict of interest between their responsibilities as care givers and their responsibilities as researchers. Finally, most IDF soldiers serve in the military under mandatory conscription laws and not necessarily of their own free will, which adds an additional protective responsibility to the military establishment. Together, these unique characteristics require that special practices be implemented in order to maintain the ethical standards mandated for all human subject research.

Military medical research is not a unique feature of the IDF. The United States Army Institute of Surgical Research (USAISR) has also explored ethical issues characteristic of military research [6]. USAISR has an advisory panel to assist researchers and decrease IRB burden, but this effort is concentrated mainly on the difference between “performance improvement” (meaning the evaluation of procedures that are part of common practice and hence do not require an IRB approval), and “research” (which is defined as the “testing of issues that go beyond medical knowledge…”) [6]. It is interesting to note that within this US context, and in contrast with the IDF, the fact that research subjects are soldiers or veterans is not considered an issue in need of special consideration. This difference may be due to the fact that the IDF is a conscription-based army, while the US army, and indeed the majority of western armies, are volunteer-based.

In this report, we aim to present the history of the IDF regulations on human research that led to the creation of the unique advisory and preparatory process (APP); describe the qualitative characteristics of the IDF’s IRB; and provide quantitative data on the IRB’s throughput and output, in order to provide a benchmark for other IRBs.

## Methods

We reviewed all relevant IDF regulations and orders pertaining to the structure, activity and oversight of the IRB and of medical research conducted in the IDF. Additionally, we analyzed the ethical review process for all research proposals submitted to the IDF APP between January 1, 2013 and December 31, 2015. We reviewed all IRB protocols for this period, and recorded specific notes and considerations cited for each IRB decision. We calculated IRB rejection rates, approval rates and revision rates, and categorized and quantified IRB requests for protocol revisions. We also calculated the APP’s effectiveness in reducing IRB burden. This study was granted an IRB waiver by the IDF medical corp’s IRB, as no personal subject data were collected or used.

## Results

### The history of the IDF MC IRB

From 1980 through the late 2000s, medical research in the IDF was regulated in accordance with Israel Ministry of Health regulations [5]. In 2007, the issue of human subject medical research in the IDF received widespread public attention in response to a television expose that reported on a previously unpublicized clinical trial conducted in the IDF between 1999 and 2005 [7]. According to this report, 716 soldiers from elite IDF units volunteered to participate in a phase II clinical study to test the efficacy of a vaccine against anthrax, in anticipation of the potential threat of this pathogen within the context of biological warfare. Several dozen of these volunteers later complained of various side effects which they attributed to having received the study vaccine [8]. Public debate surrounding the controversy focused on several key ethical issues relating to the conduct of medical research in the military setting, including the justification for recruiting soldier volunteers, the ethics of conducting confidential medical research and the ability of soldiers to provide informed consent. During the ensuing debate a petition was filed with the Supreme Court to issue an injunction to curtail all medical research in the IDF, and the Israel Medical Association submitted an amicus brief to the court suggesting that soldiers are vulnerable subjects and should therefore be disqualified from participating in medical research [9]. In 2008 the IDF Surgeon General initiated a major revision of the military orders governing medical research, in response to which the Supreme Court petition was withdrawn. To date, medical research in the IDF continues under this revised set of military regulations.

In order to protect IDF personnel within the context of human subjects research, as well as to help IDF medical researchers maintain the highest standards of ethical research, the IDF MC has established two complementary mechanisms that together form the oversight and regulatory framework that governs military medical research. The first mechanism is the IDF IRB, which functions in accordance with IDF Supreme Command Directive 2.0716 and IDF Surgeon General’s Directive 100.013 [10]. These military regulations are generally considered stricter and more conservative than the comparable guidelines that govern civilian IRBs, especially concerning on the following:

- Limitations on volunteer recruitment and recruitment setting (in cases where the study carries more than minimal risk for the solider, the researcher cannot approach the research subjects directly, and has to wait for the subjects to approach the researchers),
- Establishment of upper limits on recruitment rates,
- Prohibition of commander presence at the recruitment site,
- Burden of proof to demonstrate direct military relevance and benefit to soldier health;
- Composition of the IRB, which must include two high-level retired military personal that have substantial academic backgrounds in a non-medical topics and whose role is to try and change the research population from army-based to civilian-based.

In addition to the protective mechanisms presented above, additional safeguards have been put in place in the IDF to protect study subject welfare under special circumstances. When the study population includes soldiers in basic training, the researcher must demonstrate to the IRB that the study can be done only in this population group and that it will help this group specifically. If this criterion is not met, the IRB will not approve the study.

Since a small proportion of military medical research might be classified for security reasons, subjects entering such a study might logically be limited in their ability to consult with others regarding their ongoing participation. In order to ensure transparency and full access to consultation, current IDF regulations stipulate that all classified studies must identify an independent, non-military consulting physician to whom all study subjects must have unlimited access at all times. This independent consultant can answer any subject questions that arise before, during or after the study, and can assist subjects in reaching a decision regarding their participation and consult regarding concerns about potential side effects.

In cases where the study carries more than minimal risk for the solider, the recruitment team of the study cannot enroll soldiers directly. Rather, once the study has been presented to the target group, soldiers interested in enrolling in the study must initiate the contact with the recruitment team.

The second mechanism put in place in 2008 is the APP, which precedes the deliberation by the IRB. This preparatory process includes early guidance by the IRB secretary, medical specialists, research consultants, technical writers, and legal counsel, who together help researchers prepare for and meet the rigorous ethical, academic and scientific standards required by the IDF and its IRB.

### Review of the APP role

The fundamental idea and the most important function of the APP is to ensure the maintenance of ethical standards in IDF research protocols. However, the APP has acquired other functions over the time. First, as the APP has become a mechanism to exclude un-ethical or non-relevant proposals, it also reduces the workload of the IRB. Second, the APP assists researchers who wish to conduct their research in the IDF but do not have the knowledge of the required technical and ethical standards, by escorting the researcher from the basic research idea to a full proposal that meets IRB demands. Third, the APP assists researchers who already have a full research protocol “fine tune” it to the specific demands of the IDF IRB.

**Fig. 2:**
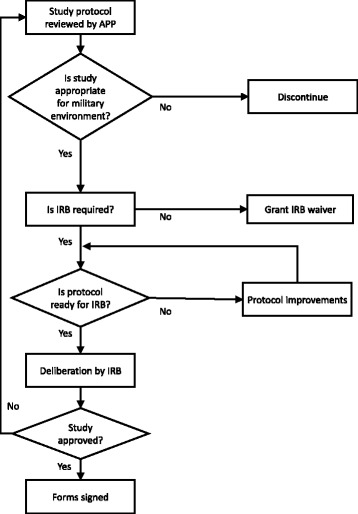
Advisory and preparatory process (APP) and Institutional Review Board (IRB) work flow algorithm

The integrative structure of the APP and its relationship with the IRB are shown in Fig. 2. It is notable that before any research protocol is deliberated by the IRB, it is sent to the APP for discussion of any potential ethical, technical and professional difficulties. Only after the APP has reviewed and approved the research protocol is it submitted to the IRB for formal deliberation. If a research protocol is not approved by the IRB, it returns to the APP to assist the investigators in making any changes or improvements that are necessary before it can be resubmitted to the IRB.

### IRB throughput and output

**Fig. 3:**
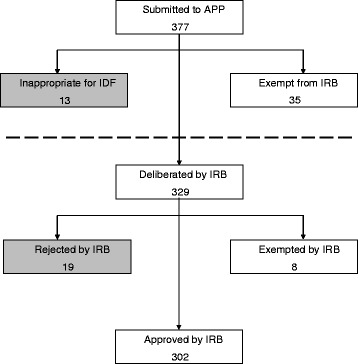
Advisory and preparatory process (APP) and Institutional Review Board (IRB) work flow and decision outcomes, 2013–2015

From 2002 through 2007, an average of 91 proposals were introduced to the IRB annually. Since the introduction of the APP in 2008 and through 2015, this annual average increased to 115. Between January 2013 and December 2015, the APP handled a total of 377 research proposals (Fig. 3). Upon initial review, 35 proposals were found to be exempt from IRB authorization and were granted waivers, while 13 study proposals were found to be inappropriate for execution in the IDF due to insurmountable flaws that precluded meeting ethical, academic or scientific standards. The remaining 329 proposals were deemed appropriate and were prepared for IRB deliberation. The IRB subsequently determined that eight of these study protocols were, in fact, entitled to waivers. 19 proposals were rejected, and the remaining 302 were authorized. Of the 377 research proposals submitted to the APP during 2013–2015, 345 (92 %) were ultimately cleared for execution, while 310 of 329 proposals (94 %) deliberated on by the IRB were cleared for execution.

Of the 302 research proposals ultimately approved by the IRB after deliberation, 134 (44 %) required 190 separate protocol revisions, of which 62 (33 %) were revisions directly associated with the additional precautions in place for military research volunteers (Table 1). The remaining 168 research proposals (56 %) were approved by the IRB with no need for protocol revisions.

**Table 1:** Distribution of institutional review board requests for protocol revisions (*N* = 134 research proposals)

Reason for revision request	Military-specific request	Number
Inadequate provisions for informed consent by soldiers	Yes	54
Inadequate justification for soldier enrollment	Yes	8
Inadequate assurance of subject anonymity	No	37
Scientific issues regarding research methods	No	44
All other	No	47
Total		190

Protocol revisions were required for various reasons. Of the 54 revision requirements related to informed consent, 40 were technical and required the investigator to add a simple, clear-cut declaration to the informed consent forms such “refusing to be a part of this study will not interfere with my military service” or “I understand that I do not have to take part in this study”. Ten revisions addressed the possibility that, during the study, the researcher might discover medical information that would require the exclusion of the soldier from his current military training assignment due to health restrictions. In these cases, the consent form was required to include an additional statement such as: “By taking part in this research I understand that new medical information about me may become available to the IDF MC, and that the discovery of such information may, under certain circumstances, jeopardize the continuity of my current military training assignment.” The remaining four informed consent revisions were related to studies that required the participant to use a medication, placebo or medical device. In these cases, the IRB required specifically tailored protocol revisions, including the following: that enrollment of mandatory service soldiers be limited to those in their final year of service; that the consent form specifically state that the study protocol requires the participant to perform a task that is not a part of his regular training and to list the added training required; and that the consent form specifically state that participating in the study will not provide the participant better accessibility to medical treatment.

Eight protocol revision requirements addressed issues relating to the method of solider enrollment. One revision required the researcher to recruit subjects from a more varied array of military units than originally proposed. Three study protocols involved the testing of defense products intended for use by both soldiers and civilians, and the IRB required a change to the enrollment ratio of these two populations. Two studies were initially intended for execution among basic trainees, but the IRB required that the enrollment be from the general military population. One revision addressed the issue of peer pressure among a specific group of soldiers targeted for enrollment, and the final revision required the appointment of an independent external consultant for a non-classified research study, due to the nature of the study topic and the target population.

Nineteen proposals were approved in the APP screening process but were eventually rejected by the IRB. Six proposals were denied due to limitations relating to data security. Four proposals were denied when the IRB became convinced that the potential risk to the enrolled solider outweighed the potential for personal benefit. One study was denied when concerns were raised that conducting it during a training course might compromise soliders’ ability to provide informed consent. Two studies were rejected on the grounds that they planned to consume a unique and limited resource best reserved for other purposes, and six were rejected as “not in the interest of the IDF MC”.

## Discussion

The unique aspects of the military environment and the ethical considerations regarding the recruitment of soldier volunteers for medical research require military IRBs to remain especially vigilant in protecting research subjects and in holding military researchers to the highest ethical standards of conduct. This is especially true for research conducted on basic trainees, who are even more dependent on their superiors, highly subject to peer pressure, and less able to provide true informed consent. Thus, in the IDF the IRB carefully parses all research proposals in order to ensure that they meet the strict military regulations regarding subject recruitment and military relevance. Recruitment rates among trainees is capped at 80 %, so that soldiers declining to provide consent for participation are “masked” by arbitrarily excluded subjects. Training cadre are excluded from recruitment meetings in order to prevent any undue influence of their presence on soldier consent. Additionally, military clinicians are prohibited from enrolling soldiers under their care as participants in clinical trials, and researchers wishing to recruit potential subjects must provide a 48 h waiting period between initial contact and signing of the consent form, in order to allow soldiers ample time to consult with family or friends.

The ultimate goal of medical research is to promote solutions to health related issues that currently lack satisfactory solutions. Since the military population and its health-related issues are inherently different from those of the civilian population, there is a real need to conduct research on this specific population, especially on topics where there is a cardinal difference between soldiers and civilians. Ethical medical research in a conscript-based army requires special consideration and care, mostly due to the soldier’s potentially limited ability to provide genuine informed consent. This principle is central to the rationale for the existence of the APP and the military IRB. For each research proposal submitted for approval in the military, the regulatory bodies must address several unique questions: Must this research be done specifically on soldier subjects? Can it be done on civilians instead? Will this research proposal ultimately benefit the soldier population? How will the investigators assure that genuine informed consent is given?

While the rationale behind the heightened vigilance of the IDF IRB is clear, it remains difficult to quantify the impact of these measures. Although technical aspects of IRB processes, such as turnaround time from proposal submission to researcher notification, have been described previously [11], there is no simple method to quantify the ethical oversight provided by institutions in the regulation of medical research [12]. A systematic review by Edwards et al. described 26 publications that compared ethical judgments across IRBs [13]. Their study found that when separate IRBs deliberated identical research protocols within the context of multi-center research trials, there was a wide variety between IRBs in the types of amendments requested (protocol revisions, patient information sheets and consent forms, recruitment method, compensation arrangements and scientific issues), but that the overall inter-IRB approval rate was consistently high in all the reviewed reports, ranging between 83 to 95 % [13–15]. It is hard to estimate the exact effect of the APP on the IDF IRB approval rate. Based on the data presented in our report, the IDF IRB approval rate without the pre-deliberation review would apparently have been 92 % (345 proposals ultimately approved or exempted, of 377 initially handled). When including the APP’s contribution to screening and excluding proposals unfit for the IRB, the IRB’s approval rate increased modestly to 94 %. This shows only the effect of the APP as a mechanism that decides if there is a need for an IRB deliberation, similar to the USAISR advisory panel. This rate is similar to the reports described in the review. However, this screening and exclusion process is only a small part of the overall APP process.

In order to address the scientific need for medical research within the military, while maintaining the requisite limitations on human subject research in the military environment, the IDF drafted and implemented specific orders and directives and founded the APP. These limitations appear to significantly challenge researchers, as 33 % of all protocol revisions requested by the IDF MC IRB were rooted in military-specific concerns regarding the proposed method for recruiting soldiers for the study or in the proposed method for obtaining their informed consent. Furthermore, the fact that just over one-third of the proposals required protocol revisions and amendments despite APP review and preparation, underscores the fervor with which the IRB protects both volunteers and researchers from potentially unethical research practices in the military environment.

There are two main limitations to the APP process. First, even though the vast majority of the initial study proposals (90 %) were amended by the APP, 47 % still required some form of additional revision when considered by the IRB. As has been previously demonstrated [12, 13, 15], different IRBs react differently to identical research protocols, and as such, it is understandable that a protocol approved by the APP might still be sent by the IRB for certain revisions. Second is the issue of time. As has been described [16], the time from proposal submission to IRB approval is a key component in the overall success of the study. The APP mechanism likely adds some time to the overall length of the process. However, the typical researcher in the IDF benefits from both ethical and technical guidance in preparing research proposals, inexperienced researchers arrive better prepared for their meeting with the IRB, and APP turnaround times are usually only several days long.

In this report we present the outcomes of all formal research proposals submitted to the APP during 2013–2015. These do not include the approximately 250 additional research initiatives that were discussed tentatively with the APP but that did not mature into formal proposals within the study window.

## Conclusions

We believe that the IDF APP mechanism has improved the scientific rigor and ethical and academic standards of IDF MC research. The pre-IRB review allows researchers to conduct medical research relevant to the military population in the IDF in a way that maintains the full integrity of soldiers’ informed consent. In addition, we hope that by sharing IRB process data and approval and rejection rates, medical institutions can help build and support a benchmarking process through which individual IRBs can appraise their own process measures relative to those of their peers.

## Abbreviations

APP: Advisory and preparatory process
IDF MC: Israel defense forces medical corps
IRB: Institutional review board
USAISR: United States army institute of surgical research

## References

[1] Hurran E (2002). Patients’ rights: from alder Hey to the Nuremberg code. History and policy.

[2] Association GAotWM (1948). WMA declaration of Geneva.

[3] WMA Declaration of Helsinki Ethical Principles for Medical Research Involving Human Subjects [database on the Internet]1968. Available from: http://www.wma.net/en/30publications/10policies/b3/index.html. Accessed 11 Nov 2014

[4] Services USDoHaH. Code of Federal Regulations - Title 45 Public Welfare CFR 46. 1981. http://www.hhs.gov/ohrp/humansubjects/guidance/45cfr46.html. Accessed 3 May 2015 2015. 11686173

[5] health Imo. Israeli ministry of health, regulation on public health. state of israel. 1981. http://www.health.gov.il/English/MinistryUnits/HealthDivision/MedicalTechnologies/Drugs/ClinicalTrials/Pages/default.aspx. Accessed 3 May 2015.

[6] Platteborze LS, Young-McCaughan S, King-Letzkus I, McClinton A, Halliday A, Jefferson TC (2010). Performance improvement/research advisory panel: a model for determining whether a project is a performance or quality improvement activity or research. Mil Med.

[7] First Press release of the IMA about the Anthrax trial Israel Medical Association. 2007. http://www.ima.org.il/ima/formstorage/type1/antrax.pdf. Accessed 22 Oct 2016.

[8] Committee IMAE (2008). the Anthrax trial - final summery.

[9] supreme court petition physician for human rights versus the minister of defence In: 9273/07. 2010. http://www.phr.org.il/%D7%9B%D7%A9%D7%9C%D7%99%D7%9D-%D7%90%D7%AA%D7%99%D7%9D-%D7%97%D7%9E%D7%95%D7%A8%D7%99%D7%9D-%D7%91%D7%A0%D7%99%D7%A1%D7%95%D7%99-%D7%A2%D7%95%D7%9E%D7%A8-2-%D7%91%D7%97%D7%99%D7%99%D7%9C%D7%99/?pr=391. Accessed 22 Oct 2016.

[10] Medical Treatment in the IDF a review. 2015. http://www.law.idf.il/163-7173-he/Patzar.aspx. Accessed 22 Oct 2016.

[11] Adams P, Kaewkungwal J, Limphattharacharoen C, Prakobtham S, Pengsaa K, Khusmith S (2014). Is your ethics committee efficient? using “IRB metrics” as a self-assessment tool for continuous improvement at the faculty of tropical medicine.

[12] Matheson LA, Huber AM, Warner A, Rosenberg AM (2012). Ethics application protocols for multicentre clinical studies in Canada: a paediatric rheumatology experience. Paediatr Child Health.

[13] Edwards SJ, Stone T, Swift T (2007). Differences between research ethics committees. Int J Technol Assess Health Care.

[14] Green LA, Lowery JC, Kowalski CP, Wyszewianski L (2006). Impact of institutional review board practice variation on observational health services research. Health Serv Res.

[15] Vick CC, Finan KR, Kiefe C, Neumayer L, Hawn MT (2005). Variation in institutional review processes for a multisite observational study. Am J Surg.

[16] Conforti L, Ross K, Hess B, Lynn L, Holmboe E, editors. Length of time needed for institutional review board approval or exemption of quality improvement projects among subset of US training programs. Academy for Healthcare Improvement Symposium. Pennsylvania: The American Board of Internal Medicine Philadelphia; 2008.

